# Why did soft drink consumption decrease but screen time not? Mediating mechanisms in a school-based obesity prevention program

**DOI:** 10.1186/1479-5868-5-41

**Published:** 2008-08-11

**Authors:** Marijke JM Chin A Paw, Amika S Singh, Johannes Brug, Willem van Mechelen

**Affiliations:** 1 EMGO-Institute and Department of Public and Occupational Health, VU University Medical Center, Amsterdam, The Netherlands; 2 EMGO-Institute, VU University Medical Center, Amsterdam, The Netherlands

## Abstract

**Objectives:**

This paper aims to identify the mediating mechanisms of a school-based obesity prevention program (DOiT).

**Methods:**

The DOiT-program was implemented in Dutch prevocational secondary schools and evaluated using a controlled, cluster-randomised trial (September 2003 to May 2004). We examined mediators of effects regarding (1) consumption of sugar containing beverages (SCB); (2) consumption of high caloric snacks; (3) screen-viewing behaviour; and (4) active commuting to school. To improve these behaviours the DOiT-program tried to influence the following potentially mediating variables: attitude, subjective norm, perceived behavioural control, and habit-strength.

**Results:**

Both in boys (n = 418) and girls (n = 436) the DOiT-intervention reduced SCB consumption (between group difference in boys = -303.5 ml/day, 95% CI: -502.4;-104.5, between group difference in girls = -222.3 ml/day, 95% CI: -371.3;-73.2). The intervention did not affect the other examined behaviours. In girls, no intervention effect on hypothetical mediators was found nor evidence of any mediating mechanisms. Boys in intervention schools improved their attitude towards decreasing SCB consumption, while this behaviour became less of a habit. Indeed, attitude and habit strength were significant mediators of the DOiT-intervention's effect (4.5 and 3.8%, respectively) on SCB consumption among boys.

**Conclusion:**

Our findings imply that interventions aimed at EBRB-change should be gender-specific. Future studies aimed at reducing SCB consumption among boys should target attitude and habit strength as mediating mechanisms. Our study did not resolve the mediating mechanisms in girls.

**Trial registration:**

International Standard Randomised Controlled Trial Number Register ISRCTN87127361

## Background

Soft drinks, fast food, and hours of computer games and television viewing are common features in today's youth. Unhealthy diets and lack of physical activity are the leading causes of avoidable illness and premature death in Europe [1]. Of particular concern is the increasingly unhealthy diet and physical inactivity of children and adolescents. The great Public Health burden of overweight and obesity requires widespread dissemination of effective prevention strategies aimed at improving energy balance related behaviours (EBRBs) (e.g. television viewing, active transport, soft drink and snack consumption) [2].

School-based interventions are promising because of their potential to reach almost all children in the population. To improve the effectiveness of interventions that aim at improving EBRBs, we need to identify how these interventions can lead to improvement of EBRB. Mediation analysis is a method to assess the processes by which an intervention achieves its intended effects [3]. Mediation analysis identifies which intermediate variables are responsible for an intervention's effects.

Most interventions are designed to change intermediate – or 'mediating'- variables that are hypothesized to be causally related to the outcome of interest. These variables are called mediators when they explain the relationship between exposure to the intervention and the outcome variable. Mediation analysis is useful, because it can be used to separate elements of an intervention that are critical to its success from those that are not. If ineffective and effective intervention elements can be identified and eliminated or expanded, respectively, an enhanced intervention program can be developed that provides greater benefit and costs less [3].

If an intervention is effective, mediation analysis can identify which mediating mechanisms are responsible for this effect. When an intervention is not effective mediation analysis can help to find possible causes of this lack of effect [3]. Maybe the intervention was not effective in influencing the mediating variables. Other competing processes may have a suppression effect – i.e. accomplish a negative intervention effect – diminishing the intervention effect caused through the mediating variables. Another possibility would be that the hypothesised mediator does not mediate behaviour change.

Improving dietary behaviour and physical activity patterns may be achieved by inducing changes in personal and environmental mediators of such EBRBs. The school-based Dutch Obesity Intervention in Teenagers (DOiT) aimed at improving the following EBRBs:

(1) consumption of sugar containing beverages, i.e. consumption of soft drinks and fruit juices;

(2) consumption of high caloric snacks, i.e. consumption of savoury and sweet snacks;

(3) screen-viewing behaviour, i.e. time spent on television viewing and computer use; and

(4) active commuting to school.

To improve these behaviours the DOiT-program tried to influence the following potentially mediating variables: attitude, subjective norm, perceived behavioural control and habit-strength. These determinants were chosen based on a combination of an analysis of systematic reviews on determinants of energy balance-related behaviours [4-6], and personal interviews with teachers, parents, experts in the field of physical activity, dietary behaviour, and behavioural change [7]. The Theory of Planned Behaviour suggests that the most proximal determinant of behaviour is the intention to perform this specific behaviour, and that three additional determinants predict the intention: attitudes, perceived subjective norms, and perceived behavioural control, or self-efficacy [8]. However, EBRBs are typically a natural part of adolescents' everyday lives that do not require much intentional effort to be set in motion [9,10]. Habitual behaviour is considered to be "automatic," triggered by environmental cues instead of conscious evaluations of possible outcomes, the opinion of other people, and confidence about being able to perform the behaviour. The Habit Strength Theory posits that when habits are formed, subsequent behaviour is automatically triggered by specific environmental cues that normally precede the action [9]. Therefore, we also included habit strength as a possible mediator of behaviour change. Earlier reports on the DOiT-study indicate that the intervention resulted in lower skin fold thickness among girls and lower consumption of sugar containing drinks among both boys and girls [11].

There have been few formal mediational analyses conducted for school-based physical activity and nutrition intervention programs. To our knowledge, no studies are available on mediating variables in interventions specifically aimed at reducing time spent in screen-viewing behaviour, or consumption of soft drinks or high-energy snacks.

The purpose of this study was to examine whether the DOiT-program was effective in improving the targeted mediators and to identify the mediating mechanisms targeted by the DOiT-program.

## Methods

### Study design and participants

The DOiT-study was a cluster randomised controlled trial, with measurements at baseline, after eight, twelve, and twenty months. We recruited for participation in the trial 18 prevocational secondary schools located at a maximum of 150 kilometres from Amsterdam. We restricted invitation to schools of the lowest educational level, since an inverse relationship exists between educational level/socio-economic status and prevalence of obesity in many western countries [12]. All participating schools selected three classes of first-year students (aged 12–13 years), who received an informational brochure about the study. The selection of classes was based on practical reasons (e.g. similar timetables for lessons physical education). Randomisation took place at the school level (10 intervention and 8 control schools) and was stratified by degree of urbanization, using SPSS statistical software (SPSS Inc, Chicago, Ill). All participating students and their parents gave written informed consent. The Medical Ethics Committee of the VU University Medical Center approved the study protocol.

### Intervention

We developed the DOiT-program applying the Intervention Mapping protocol, which facilitates a systematic process of designing health promotion interventions. Intervention Mapping is based on theory and empirical evidence [13]. Concisely put, the three key input elements within IM are (1) a careful literature search for empirical findings, (2) the assessment and use of theory, and (3) the collection of new data. The IM protocol consists of five steps: (1) the definition of program objectives, based on a thorough analysis of the health problem, (2) the selection of adequate theories and methods to realize behavioural change, (3) the design of the intervention program, as well as the selection, testing, and production of the intervention materials, (4) the development of a plan for the implementation, and (5) evaluation. The development and content of the DOiT-program are described in more detail elsewhere [7]. DOiT was based on the EnRGframework [10], an integrative framework that applies insights from Dual-Process Theory [14], the ANGELO model [15], the Theory of Planned Behaviour [8], and habit theory [9].

**Figure 1:**
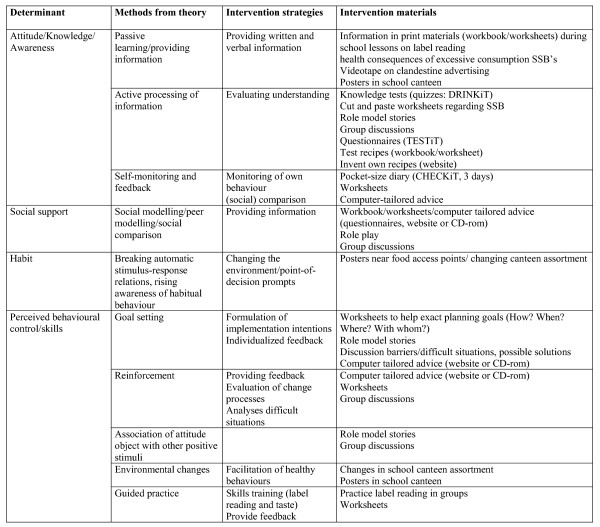
**Intervention materials and strategies used to change each of the hypothesized determinants in the DOiT-intervention.** Example: determinants of reduction consumption of sugar-sweetened beverages.

**Figure 2:**
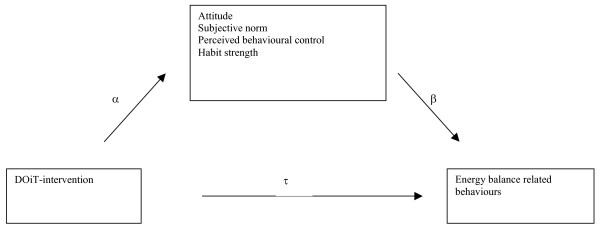
**Conceptual mediational model: The DOiT program affects energy balance related behaviours indirectly through mediator variables**.

The DOiT-program was hypothesized to change the following mediators: attitude, subjective norm, behavioural control, habit strength concerning specific EBRBs. Figure 1 shows an example of intervention materials and strategies used to change each of the hypothesized determinants. In turn these mediators would produce changes in (1) consumption of sugar containing beverages, i.e. consumption of soft drinks and fruit juices; (2) consumption of high caloric snacks, i.e. consumption of savoury and sweet snacks; (3) screen-viewing behaviour, i.e. time spent on television viewing and computer use; and (4) active commuting to school (see figure 2). The intervention consisted of an individual component and an environmental component. The individual component consisted of an educational program covering eleven lessons for the subjects biology and physical education. The environmental component involved encouraging additional physical education classes and changes at school cafeterias. Control schools were requested to maintain their regular curriculum.

### Measurements

A trained research team completed all measurements according to a standardized protocol. The research assistants were also involved in the organization of the measurements, and were therefore not blinded to group assignment. The current study includes the measurements at baseline and immediately post-intervention (after eight months).

#### Anthropometrics

Body height was measured and recorded with a portable stadiometer (Seca 225; Seca Deutschland Hamburg, Germany) with an accuracy of 1 mm. Body weight was measured and recorded within 0.1 kg using a calibrated electronic flat scale (Seca 888; Seca Deutschland). Body Mass Index was calculated as weight in kilograms divided by the square of height in meters, and using the cutoff values as described by Cole et al [16].

#### Energy balance related behaviours

The DOiT questionnaire was based on other validated questionnaires for assessing dietary intake, physical activity, behaviour-specific cognitions, and habit strength [17,18]. We adjusted the questions making them relevant to our study population (e.g. active commuting to school instead of work). The structure of the questionnaire was equal for all energy balance related behaviours: (1) consumption of sugar containing beverages, i.e. consumption of soft drinks and fruit juices; (2) consumption of high caloric snacks, i.e. consumption of savoury and sweet snacks; (3) screen-viewing behaviour, i.e. time spent on television viewing and computer use; and (4) active commuting to school. For example, adolescents had to indicate how many days a week they consumed sugar containing beverages, and the amount/number of servings of sugar containing beverages they usually consumed on these days, thinking of the last week. For TV viewing adolescents had to indicate on how many days they watched television in the last week. On days they watched television, adolescents had to indicate how long on average they watched television, thinking about a usual school day. Frequency and quantity were multiplied to obtain estimates of mean daily consumption or mean daily screen-viewing behaviour or active transport.

#### Mediators

Behavioural specific questions on personal and social environmental determinants of each of the risk behaviours included questions on attitude, subjective norm, perceived behavioural control, and habit strength. Most variables were measured on bipolar five-point Likert scales anchored by -2 and +2.

#### Attitude

Adolescents' attitude about reducing their television viewing, sugar containing beverage and snack consumption, and increasing their physical activity was assessed by five items that consisted of beliefs about the consequences of improving the specific EBRB (e.g. "If I watch less television during leisure time, 'I would be much more fit' or 'I would be much more bored'). The responses were summed into a sum-score ranging from -10 to +10, with a higher score indicating a more positive attitude about improving the specific behaviour. The attitude scales showed good internal consistency (Cronbach's α ranging from 0.72–0.94).

#### Subjective norm

Six items on encouragement and support for improving each EBRB were included: 'My parents think I should drink less sugar containing beverages' (yes, certainly...no certainly not), 'Do your friends drink SCB?', 'Do your parents encourage you to reduce your sugar containing beverage consumption' (yes a lot, no not very often). The responses were summed into a sum-score ranging from -12 to +12, with a higher score indicating more encouragement and support for improving the specific behaviour. The subjective norm scales showed good internal consistency (Cronbach's α ranging from 0.79–0.89).

#### Perceived behavioural control

Perceived behavioural control included five items that pertained to the perceived ability to improve each specific EBRB in a variety of situations (e.g., 'Do you think you could eat less snacks at school?/during television viewing?/with friends?') with answers ranging from 'No, certainly not' (-2) to 'Yes, certainly' (+2). The responses were summed into a sum-score ranging from -10 to +10, with a higher score indicating higher perceived behavioural control towards improving the specific behaviour. The perceived behavioural control scales showed good internal consistency (Cronbach's α ranging from 0.80–0.91).

#### Habit strength

Adolescents' were asked to what extent they agreed (+2) or disagreed (-2) to three different statements regarding each specific EBRB: 'Walking/biking somewhere is something I do often; is something I do automatically; is something that fits me.' The responses were summed into a sum-score ranging from -6 to +6, with a higher score indicating stronger habit. The habit scales showed good internal consistency (Cronbach's α ranging from 0.82–0.96).

### Statistical analyses

Analyses were performed for boys and girls separately, since gender appeared to be an effect modifier or moderator. Thus gender-specific mediators were sought. Multilevel linear analysis (MLwiN version 2.02) was performed to determine whether there were baseline differences in EBRBs and hypothetical mediators between the intervention and control group. Using this technique, standard errors of regression coefficients can be adjusted for the dependency in observations within one school and/or class. We defined three levels in our multi-level analysis: 1) student, 2) class, and 3) school.

The mediation analysis was also performed using multilevel linear analysis (MLwiN version 2.02). First, we calculated the direct effect of the DOiT-intervention on the behavioural outcome measures (τ). In this regression model the behavioural outcome value at 8 months was adjusted for the baseline value. Second, we calculated the effect of the intervention on the theoretical mediator, adjusted for baseline values (α). We computed the residualised change score of the mediators. The residualised change score is the post-intervention score, adjusted for the pre-intervention score and therefore represents change adjusted for baseline values. Third, we calculated the effect of the residualised change score of the hypothetical mediator on the outcome, after controlling for the intervention and the behavioural baseline scores (β). The mediating effect is the product of the α and β-values (α*β) and provides an estimate of the magnitude of the mediation effect in the units of the outcome variable. To test for mediation, we used the products of coefficient method [19]. This method assesses the statistical significance of a mediating effect by dividing the products of the coefficients α and β by its standard error (SEαβ = √(α^2^*SEβ^2 ^+β^2^*SEα^2^). Finally, we calculated the percentage of the program effect that was attributable to each of the mediational pathways. The numerator represents the value of the mediating effect (α*β). The denominator represents the unadjusted direct effect of the DOiT-intervention on the behavioural outcome measures (τ). Complete mediation refers to a situation where the intervention effect can be totally explained by the mediator. When the intervention can be partially explained by the mediator this is partial mediation. When statistical removal of a meditational effect increases the magnitude of the relationship between the intervention and the outcome indicates suppression [3].

## Results

### Baseline characteristics

Of 1323 invited adolescents, 1108 (84%) returned both the parental and student informed consent. In total 1053 students were measured at baseline while 1031 (98%) were measured at 8-month follow-up. Table 1 shows the baseline characteristics of the study sample with complete data (n = 854), stratified for gender. In girls, the mean age was 12.6 years and 16% was classified as overweight and 3% as obese. In boys, the mean age was 12.8. In the intervention group 10% of the boys was overweight and 2% obese, while in the control group the prevalence of overweight and obesity was 15 and 2% respectively (between-condition difference p = 0.01).

**Table 1:** Demographic and anthropometric characteristics at baseline in intervention and control schools.

	**girls**		**boys**	
**Characteristics**	**intervention** **group** ** (n = 248)**	**control** **group** ** (n = 188)**	**intervention** **group** ** n = (213)**	**control** **group** ** (n = 205)**

**mean (sd) age, y**	12.6 (0.4)	12.7 (0.5)	12.7 (0.5)	12.8 (0.5)
**ethnicity, No. non western (%)**	29 (12)	21 (11)	25 (12)	34 (17)
**mean (sd) BMI, kg/m**^2^	19.0 (3.0)	19.5 (3.4)	19.0 (2.6)	19.3 (2.9)
**overweight, No. (%)**^**a**^	35 (14)	33 (18)	21 (10)*	62 (15)
**obese, No. (%)**^**a**^	8 (3)	7 (4)	4 (2)	8 (2)

^a ^using cut-off values described by Cole et al. [16].

* significantly different from the control group (p = 0.01)

### Intervention effects on EBRBs

Table 2 shows the baseline and post-intervention values of the energy-balance related behaviours. At baseline, the boys and girls in the intervention group reported spending significantly more minutes on active transport (42 and 40 min/wk, respectively) than boys and girls in the control group (34 and 34 min/wk, respectively). At baseline, girls in the control group reported a higher consumption of sugar containing beverages than girls in the intervention group (1121 versus 1073 ml/day). Both in boys and girls we observed a significant intervention effect on consumption of sugar containing beverages (between group difference in boys = -303.5 ml/day, 95% CI: -502.4;-104.5, between group difference in girls = -222.3 ml/day, 95% CI: -371.3;-73.2) (Tables 5 and 6). The intervention did not significantly affect the other EBRBs. Therefore, a mediation analysis was only performed for the effect on consumption of sugar containing beverages.

**Table 2:** Energy balance-related behaviours at baseline and post-intervention in intervention and control schools, means (SD)

		**girls**			**boys**	
		**baseline**	**post-** **intervention**		**baseline**	**post-** **intervention**
**screen-viewing behaviour** ** (min/day)**						
control	(n = 181)	247 (136)	247 (149)	(n = 196)	288 (145)	263 (140)
intervention	(n = 238)	215 (119)	206 (135)	(n = 206)	271(159)	244 (150)
**active transport to school** ** (min/wk)**						
control	(n = 179)	34 (27)	42 (27)	(n = 188)	34 (27)	38 (26)
intervention	(n = 185)	40 (29)*	46(29)	(n = 185)	42 (30)*	45 (29)
**sugar-containing beverage** ** consumption (ml/day)**						
control	(n = 165)	1121 (807)	1010 (693)	(n = 180)	1186 (933)	1184 (856)
intervention	(n = 218)	1073 (777)*	739 (564)	(n = 190)	1107 (919)	826 (749)
**high caloric snack** ** consumption (portion/day)**						
control	(n = 180)	1.9 (1.3)	1.9 (1.3)	(n = 184)	2.1 (1.5)	2.1 (1.3)
intervention	(n = 238)	1.9 (1.3)	2.0 (1.5)	(n = 191)	2.0 (1.4)	2.0 (1.5)

* significantly different from the control group (p ≤ 0.02)

### Intervention effects on mediators

Tables 3 and 4 show the baseline and post-intervention values of the hypothesized mediators for boys and girls, respectively. At baseline, the intervention and control schools were comparable regarding the hypothetical mediator variables. Except for perceived behavioural control regarding decreasing the amount of sugar containing beverages, which was higher in girls from the intervention schools. In girls the DOiT-program was not effective in influencing the hypothesized mediators (Table 6). In boys, the DOiT-program significantly improved the subjective norm regarding increasing active transport and decreasing snack consumption. Furthermore, the DOiT-intervention improved attitude and decreased habit strength regarding sugar containing beverage consumption (Table 5).

**Table 3:** Determinants of energy balance-related behaviours at baseline and post-intervention among boys from intervention and control schools, means (SD)

		**intervention**			**control**	
	n	baseline	post- intervention	n	baseline	post- intervention
**screen-viewing behaviour**						
- attitude	201	-0.7 (1.8)	-0.5 (2.0)	189	-0.6 (1.6)	-0.6 (1.5)
- subjective norm	198	-3.4 (3.3)	-3.3 (3.7)	190	-3.3 (3.2)	-3.1 (3.4)
- perceived control	192	-0.6 (5.1)	-0.3 (4.8)	189	-1.2 (4.8)	-0.5 (4.7)
- habit	205	1.0 (2.9)	0.5 (2.9)	194	1.2 (2.7)	1.0 (2.8)
**active transport to school**						
- attitude	192	2.8 (2.5)	2.6 (2.7)	186	2.4 (2.5)	2.0 (2.6)
- subjective norm	203	-3.3 (3.2)	-2.2 (3.7)	189	-3.1 (3.2)	-3.0 (3.3)
- perceived control	200	2.7 (3.8)	2.5 (3.8)	190	3.2 (3.7)	2.7 (3.9)
- habit	203	2.2 (2.5)	1.9 (2.6)	191	2.2 (2.3)	2.0 (2.4)
- perceived environment	202	-0.03 (1.7)	0.2 (1.5)	197	0.2 (1.5)	0.1 (1.5)
**sugar-containing beverage consumption**						
- attitude	171	0.6 (1.8)	0.7 (2.3)	181	0.7 (2.2)	0.2 (2.5)
- subjective norm	163	-3.8 (2.8)	-3.6 (3.4)	167	-3.7 (3.3)	-4.0 (3.2)
- perceived control	173	2.3 (4.2)	1.7 (4.4)	182	1.8 (4.5)	1.2 (4.5)
- habit	177	0.2 (2.7)	0.1 (3.0)	180	0.6 (2.8)	1.0 (2.9)
**high caloric snack consumption**						
- attitude	202	0.8 (2.4)	1.1 (2.6)	188	1.2 (2.5)	1.1 (2.3)
- subjective norm	198	-3.3 (3.3)	-2.2 (3.3)	190	-2.8 (3.4)	-2.8 (3.2)
- perceived control	203	1.8 (4.3)	2.0 (4.0)	197	1.7 (3.8)	1.8 (3.5)
- habit	205	-0.3 (2.6)	-0.5 (2.5)	196	-0.4 (2.6)	-0.4 (2.5)

* significantly different from the control group (p = 0.05)

**Table 4:** Determinants of energy balance-related behaviours at baseline and post-intervention among girls from intervention and control schools, means (SD)

		**intervention**			**control**	
	n	Baseline	post- intervention	n	baseline	post- intervention
**screen-viewing behaviour**						
- attitude	232	-0.5 (1.6)	-0.6 (1.9)	173	-0.4 (1.7)	-0.8 (1.9)
- subjective norm	227	-3.9 (3.2)	-3.6 (3.1)	175	-3.2 (3.2)	-3.2 (3.6)
- perceived control	232	-0.4 (4.3)	-0.5 (4.7)	173	-1.3 (4.6)	-1.7 (4.5)
- habit	241	0.8 (2.7)	0.8 (3.0)	179	1.1 (2.5)	1.3 (2.7)
**active transport to school**						
- attitude	222	2.7 (2.3)	3.1 (2.7)	172	2.7 (2.2)	3.0 (2.7)
- subjective norm	237	-2.8 (3.2)	-2.6 (3.0)	179	-2.5 (3.1)	-3.0 (3.2)
- perceived control	234	2.7 (3.2)	2.4 (3.8)	180	2.7 (3.3)	2.4 (3.6)
- habit	240	2.0 (2.1)	1.9 (2.4)	182	1.9 (2.1)	2.0 (2.1)
- perceived environment	240	0.1 (1.4)	0.2 (1.5)	183	-0.04 (1.2)	0.07 (1.3)
**sugar-containing** ** beverage consumption**						
- attitude	206	1.4 (2.3)	1.6 (2.8)	175	1.2 (2.3)	1.4 (2.5)
- subjective norm	202	-3.6 (2.9)	-4.3 (3.3)	172	-3.7 (3.2)	-4.2 (3.4)
- perceived control	206	1.9 (4.3)	1.6 (4.6)	173	1.7 (4.6)*	1.7 (4.6)
- habit	210	0.3 (2.6)	-0.2 (2.9)	171	0.3 (2.7)	0.2 (2.6)
**high caloric snack consumption** ** consumption**						
- attitude	230	1.9 (2.1)	1.9 (2.4)	172	1.8 (2.0)	2.0 (2.6)
- subjective norm	221	-2.9 (3.2)	-2.6 (3.2)	175	-2.8 (3.4)	-2.8 (3.2)
- perceived control	233	1.8 (4.0)	1.4 (4.0)	180	1.7 (3.3)	1.3 (3.8)
- habit	237	-0.2 (2.6)	-0.03 (2.6)	180	-0.3 (2.2)	-0.2 (2.4)

* significantly different from the control group (p = 0.05)

**Table 5:** Intervention effects on energy balance related behaviours, mediators, mediator effects, 95% confidence intervals and percent mediation among boys

	**intervention effect on** **outcome** **(τ)** ** (95% CI)**	**intervention** **effect on** **mediator** ** (α)**	**mediator** **effect on** **outcome** ** (β)**	**mediated effect** (α*β) ** (95%CI)**	**%** ** mediation**
**screen viewing behaviour** ** (min/day)**	-13.4 (-39.9 ;13.5)			NA	NA
• attitude		0.1	-13.0		
• subjective norm		-0.1	5.2		
• perceived control		-0.1	-17.2*		
• habit		-0.4	40.1*		
**active transport to school** ** (min/wk)**	1.1 (-5.4 ;7.5)			NA	NA
• attitude		0.56	-2.0*		
• perceived environment		0.15	0.0		
• subjective norm		0.78*	-0.1		
• perceived control		0.26	0.37		
• habit		0.1	0.4		
**sugar-containing beverage** ** consumption (ml/day)**	-303.5 (-502.4;-104.5)*				
• attitude		0.6*	-152.3*	-89.4 (-176.4;-2.4)*	4.5
• subjective norm		0.5	-35.4	-15.8 (-63.8;32.2)	suppression
• perceived control		0.3	-122.7*	-30.3 (-133.5;72.9)	suppression
• habit		-0.6*	155.8*	-92.7 (-184.8;-0.6)*	3.8
**high caloric snack** ** consumption (portion/day)**	-0.0 (-0.3;0.3)			NA	NA
• attitude		0.1	-0.3*		
• subjective norm		0.7*	-0.0		
• perceived control		0.2	-0.3*		
• habit		-0.1	0.2*		

* (p < 0.05).

NA = not applicable

**Table 6:** Intervention effects on energy balance related behaviours, mediators, mediator effects, 95% confidence intervals and percent mediation among girls

	**intervention effect on** **outcome** **(τ)** ** (95%CI)**	**intervention** **effect on** ** (α)**	**mediator ** **effect on** **outcome** ** (β)**	**mediated effect** (α*β) ** (95%CI)**	**%** ** mediation**
**screen viewing behaviour ** **(min/day)**	-18.5 (-49.0 ;12.0)			NA	NA
• attitude		0.2	-16.0*		
• subjective norm		-0.5	13.1*		
• perceived control		0.6	-34.3*		
• habit		-0.3	44.7*		
**active transport to school** ** (min/wk)**	-2.8 (-9.6 ;4.0)			NA	NA
• attitude		0.1	2.6*		
• perceived environment		0.1	0.1		
• subjective norm		0.3	-1.6		
• perceived control		-0.1	1.6		
• habit		-0.1	1.2		
**sugar-containing beverage** ** consumption (ml/day)**	-222.3 (-371.3;-73.2)*				
• attitude		0.1	-126.4*	-9.5 (-70.6;51.6)	suppression
• subjective norm		-0.1	-15.6	1.9 (-10.2;13.9)	8.5
• perceived control		-0.4	-105.8*	37.3 (-73.2;147.7)	suppression
• habit		-0.3	124.2*	-34.3 (-95.2;26.6)	10.0
**high caloric snack** ** consumption (portion/day)**	0.2 (-0.2 ;0.5)			NA	NA
• attitude		-0.2	-0.4*		
• subjective norm		0.3	0.1		
• perceived control		0.1	-0.4*		
• habit		0.2	0.4*		

*p < 0.05).

NA = not applicable

### Mediation

In Table 5 and Table 6 we present the intervention effects on the outcomes (τ) and hypothetical mediators (α), and the effect of the mediator on the outcome (β) for all four EBRBs for boys and girls, respectively. Since we only found a significant intervention-effect on sugar containing beverage consumption we only computed the mediated effect (α*β) and the percentage mediation for sugar containing beverage consumption.

In boys, a small but significant mediation effect of attitude towards decreasing sugar containing beverage consumption (4.5%) and habit strength regarding sugar containing beverage consumption (3.8%) was found. This mediation effect was partial. Changes in some potential mediators were significantly predictive of behaviour change, irrespective of whether participants were exposed or not to the intervention including:

- perceived control and habit strength regarding reducing screen viewing behaviour;

- subjective norm regarding active transport;

- attitude, perceived behavioural control and habit strength regarding reducing sugar containing beverage consumption and high caloric snack consumption.

In girls, none of the hypothetical mediators appeared to mediate the intervention effect. Changes in some potential mediators were significantly predictive of behaviour change, irrespective of whether participants were exposed or not to the intervention including:

- attitude, subjective norm, perceived control and habit strength regarding reducing screen viewing behaviour;

- attitude regarding active transport;

- attitude, perceived behavioural control and habit strength regarding reducing sugar containing beverage consumption and high caloric snack consumption.

## Discussion

The purpose of this paper was to examine whether the DOiT- program was effective in improving the targeted mediators – i.e. attitude, subjective norm, perceived behavioural control, and habit strength – and to identify the mediating mechanisms targeted by the DOiT-program. In girls, the DOiT-program was ineffective in changing the hypothesized mediators, while in boys the DOiT-program improved some mediators. Although changes in many of the hypothesized mediators were significantly related to behaviour change, only attitude towards decreasing sugar containing beverage consumption (4.5%) and habit strength regarding sugar containing beverage consumption (3.8%) partially mediated the intervention effect in boys.

The DOiT-intervention was effective only in decreasing sugar containing beverage consumption, and not in improving the other targeted EBRBs. Sugar containing beverage may be easier to change, since there are relatively easy available alternatives such as light versions of soft drinks, and water. Furthermore, since baseline consumption was quite high there was substantial room for improvement. This was not the case for the other EBRBs: Participants reported to eat two high caloric snacks per day, leaving only two change options a day; the majority of the children already cycled to school, leaving little room for improvement. Unfortunately, we have no information on active transport to other locations.

Our findings imply another explanation for the lack of effect on active transport, screen time and snack consumption: the DOiT-program was ineffective in changing the hypothesized mediators of these EBRBs. The DOiT-program was only effective in changing some of the mediators and only among boys. Another possibility is that the questionnaire was not sufficiently sensitive to detect changes in these mediator variables. Due to the multi-component nature of the intervention we cannot conclude which components of the DOiT-program were effective and which were not. An explanation for the small mediating effect on sugar containing beverages may be that other variables – such as environmental change options – were partly responsible for this intervention effect.

### Comparison with other studies

Our finding that intervention effects were different for boys and girls is in line with previous school-based interventions from Gortmaker et al. [20] and Flores et al. [21].

There have been few formal mediational analyses conducted for school-based physical activity and nutrition intervention programs. Haerens et al [22] found that self-efficacy was a partial mediator in changing total and school-related physical activity among adolescents in a school-based intervention in middle schools with parental support. However, the suppressor effect on attitude decreased this effect. Dishman et al. [23,24] found that self-efficacy and enjoyment partially mediated the positive effect on physical activity of a school-based intervention to increase physical activity and fitness among adolescent black and white girls. To our knowledge, no studies are available on mediating variables in interventions aimed at reducing time spent in screen-viewing behaviour, or consumption of soft drinks or high energy snacks. Haerens et al. [25] investigated the mechanisms through which a school-based fat intake intervention in adolescent girls was effective. They concluded that none of the examined hypothesized mediating variables of low fat intake were identified as mediators of changes in fat intake.

### Limitations

Study limitations include the measurement of the mediators and EBRBs. The data collection of both relied exclusively on self-report. Social desirability bias and over- and underreporting biases may have influenced the measurement of EBRBs. Since there are no validated Dutch questionnaires focussing on the mediators and EBRBs that we measured in our trial, we adapted existing validated questionnaires. Our questionnaire was not tested on validity, reliability or responsiveness. The questions aimed to assess attitude, subjective norm, perceived behavioural control and habit strength regarding *changing *four specific EBRBs. Change in behaviour may be a more difficult construct than the behaviour itself. Furthermore, if the students had changed their behaviour as a result of the intervention, asking the same question again post intervention would be a different question, namely attitude regarding changing a different behaviour. This complicates finding a change in the mediators.

Another limitation is the simultaneous measurement of hypothetical mediators and behavioural outcomes. Logically, any change in a mediator must precede a change in the outcome. As in many randomised control trials we assessed the hypothetical mediators at the same time points as the behavioural outcomes. Therefore, our statistical analysis of mediating mechanisms is correlational and cannot establish causality. To establish definitively that improved attitude towards behaviour change and reductions in habit strength are causal mechanisms in reducing SCB consumption would require a subsequent intervention study in which both are directly manipulated. Nonetheless, our results suggest that in boys the DOiT-program may have achieved its effect on sugar containing beverage consumption in part by changing attitude and habit strength.

### Strengths

Strengths of our study are the high compliance rate among adolescents and the focus on adolescents from a lower educational level. Moreover, this is one of the few studies on mediation in school-based obesity prevention interventions and to our knowledge the first study looking at a potential mediating effect of habit strength.

## Conclusion

Our findings imply that interventions aimed at reducing sugar containing beverage consumption that are developed gender-specific may be more effective. Interventions aimed at reducing sugar containing beverage consumption among boys may be more effective when focussing on modification of attitude and habit strength. For girls, apparently other mediating mechanisms were responsible for the reduction in sugar containing beverage consumption.

## Competing interests

The authors declare that they have no competing interests.

## Authors' contributions

MC, AS, JB, and WvM participated in the design of the study and contributed intellectual input into the main ideas of this paper. MC performed the statistical analysis and drafted the manuscript. AS coordinated the implementation of the intervention and supervised the data-collection. All authors read and approved the final manuscript.
